# Provision and awareness for isoniazid preventive therapy among PLHIV in Addis Ababa, Ethiopia

**DOI:** 10.1186/1472-698X-12-2

**Published:** 2012-03-27

**Authors:** Amenu Wesen, Getnet Mitike

**Affiliations:** 1 Consultant at World Health Organization, South Sudan, Africa; 2 School of Public Health, Medical Faculty, Addis Ababa University, Addis Ababa, Ethiopia

**Keywords:** HIV/AIDS, IPT, TB

## Abstract

**Background:**

The risk of acquiring tuberculosis by People living with HIV (PLHIV) could significantly be reduced through provision of isoniazid preventive therapy (IPT). In Ethiopia, it is neither practiced well nor researched in depth. Our objective was to assess IPT provision and awareness among PLHIV in Addis Ababa City Administration.

**Methods:**

Between February 2008 and May 2008, a cross sectional facility-based survey was conducted by exit interview of 406 PLHIV from six health facilities. The findings were analyzed and described in this report.

**Results:**

The proportion of PLHIV ever had been provided with IPT were 74 of 231 TB free PLHIV (32.0%) and the proportion of having information about IPT among study participants was 29.8%. Females were about two times more informed about the provision of IPT in their health facilities than males [AOR (95%CI): 2.18 (1.31-3.61)].

**Conclusions:**

We conclude that the practice of provision of IPT for PLHIV is high, but there is room for improvement. Provision of INH for TB free PLHIV has to be strengthened with better diagnostic facilities to certainly rule out active TB cases.

## Background

The spread of the HIV epidemic throughout sub-Saharan Africa has been accompanied by up to a fourfold increase in the number of TB cases registered by national TB programs [1]. Several researchers have reported the benefit of IPT for PLHIV in reducing the incidence of tuberculosis including in resource poor countries where TB infection rates are very high [2-7]. Interventions for preventing and treating TB include: IPT intensified case finding for active TB and TB infection control [8]. The World Health Organization recommends IPT for all PLHIV in countries with a prevalence of latent TB infection > 30%, and for all PLHIV with documented latent TB infection or exposure to an infectious TB case. In the same policy report it is indicated that the combined use of IPT and antiretroviral therapy among PLHIV significantly reduces new infections [8]. Most IPT studies focused on issues related to adherence by PLHIV [9,10].

Information about the status of IPT provision and awareness of PLHIV regarding IPT in Ethiopia is limited. This is due to the absence of well standardized information flow at all levels and scarcity of research based evidences.

Therefore, this paper attempted to present the status of IPT provision and awareness among PLHIV in Addis Ababa City Administration.

## Methods

### Design

This is facility-based cross sectional descriptive study which was conducted at one referral hospital and five health centers in Addis Ababa, Ethiopia.

### Setting

The study setting include Zewditu Memorial hospital which is one of the HIV therapy and care hospital and five health centers namely: Bole, T/Haymanot, Gulele, Kazanchis and Woreda 23 health centers to assess the status of IPT provision and awareness among PLHIV attending HIV chronic care clinics. Fifty eight percent of all health facilities in the city provide TB/HIV collaborative services.

### Participants

The study participants include people living with HIV and chronic follow up in the health facilities.

### Sampling method

Using a single population proportion formula with assumption of 50% prevalence of IPT practice in the country, 5% margin of error and adding a 5% allowance for interruption of participation, a sample of 403 study participants was calculated. Randomly selected PLHIV attending HIV chronic care clinics at the selected health facilities in their follow-up care were interviewed at exit. To allocate the number of PLHIV required to interview at each facility, sampling with Proportional allocation to their HIV patient load was applied.

### Data collection

The data collection was conducted from February to May 2008. Three senior VCT counselors conducted the interviews and the principal investigator provided supportive supervisions during data collection by checking completeness and consistency of questionnaires. The interviewers were recruited from health facilities other than the study sites for minimizing interviewer bias. We used interviewer administered questionnaire, which was adapted from WHO guidelines prepared for monitoring and evaluation of TB/HIV activities. As the questionnaire extracted from WHO standard guideline which is the prime source for national guideline for implementation of TB/HIV activities in Ethiopia, its validity and reliability is acceptable.

Ethical clearance was obtained from Institutional Review Board of Addis Ababa University and Addis Ababa City Administration Health bureau. Permission was obtained from authorities of the respective health facilities. The interview was undertaken after ascertaining written consents from study participants. The questionnaire did not include any identifier of the interviewee.

### Data analysis

SPSS version 11 was used for data entry and analysis. Data was analyzed using frequency distribution and characterization of the study population and then logistic regression was applied to identify factors associated with access to IPT as a package of care.

## Results

A total of 406 PLHIV, who were under HIV chronic care follow-up in government health facilities in Addis Ababa City Administration, participated in the study. Out of the total respondents 252(62.1%) were females.

The mean age (± SD) of the study population was 36.27 (± 8.74) years. Three hundred and nine (76.1%) of the participants were in the age group 25-44, 154 (37.9%) were married and 128 (31.5%) were singles, 374 (92.1%) were followers of Christian religion. Fifty two percent had completed high school, 14.5% had at least a certificate after completing high school. The remaining 17.2% and 16.5% accounted for primary completed and having no formal education at the time of the study respectively.

One hundred twenty four (30.5%), ninety seven (23.9%) and sixty eight (16.3%) were privately employed, housewives and government employees by occupation respectively; whereas 93 (22.9%) of study participants were unemployed at the time of data collection.

Close to 85% of all study participants had become more than six months since they know their positive HIV status. Three hundred and thirty two (81.8%) of these clients have already started ART in the health facilities. Of those put under highly active antiretroviral therapy (HAART), 188 (46.3%) were on ART for more than one year.

### Awareness about IPT

The proportion of PLHIV and without active TB who have some information about IPT was 29.8% (Table 1).

**Table 1:** Disease specific characteristics of people living with HIV at HIV chronic care clinics of public facilities in Addis Ababa City Administration, February, 2008.

Variable	Number	Percent
**Ever been provided with IPT, n = 231 *(free from active TB)***		
Yes	74	32.0
No	157	68.0
**Awareness about IPT**		
Yes	121	29.8
No	285	70.2
**Knowledge about IPT service availability in health facilities**		
Yes available	104	25.6
Not available	169	41.6
I don't know	133	32.8
**If the response to knowledge to IPT is not available, the reasons mentioned were. (n = 169)**		
I don't know	74	43.8
Care takers don't order	92	54.4
Other reasons	3	1.8
**Placement of the drug for IPT to be collected, (n = 74)**		
HIV clinic	64	86.5
TB clinic	1	1.4
Work place	9	12.2
**Length of being diagnosed for HIV positive**		
Less than 6 months	60	14.8
6 months-one year	87	21.4
More than one year	259	63.8
**Length of being on HAART**		
Less than 6 months	70	17.2
6 months- one year	74	18.2
More than one year	188	46.3
Not yet started	74	18.2

n = 406

Multiple logistic regression analysis with stepwise selection was used to examine potential effects of the socio-demographic variables including age, sex, educational level, marital status, occupation and other variables on knowledge of access to IPT as a package of care for PLHIV. Females (29.7%) were more likely to be informed than males (18.8%) in pertaining to the provision of IPT in their health facilities for those PLHIV having no active TB [AOR (95%CI): 2.18 (1.31-3.61)].

In addition, those who completed secondary (27.6%) and post secondary (30.5%) education were 2.5 and 3.5 times more likely than those non formally educated (14.9%) to be informed on the availability of IPT [AOR (95%CI): [2.55 (1.19-5.43), 3.47 (1.37-8.78)] respectively.

Information about the availability of IPT as a package of care among PLHIV in the different health facilities did not reveal statistically significant difference (Table 2).

**Table 2:** Association of selected variables with Awareness about access to IPT as a package of care among PLHIV, Addis Ababa City Administration, February, 2008.

Variable	Access to IPT as a Package of Care		Crude Odds Ratio (95%CI)	Adjusted Odds Ratio (95%CI)
			
	Yes (104)	No (302)		
**Sex**				
Male	29	125	1.00	1.00
Female	75	177	1.83(1.12, 2.97)	**2.18(1.31, 3.61)**
**Educational Status**				
No formal education	10	57	1.00	1.00
Primary (1-6)	18	52	1.97(0.84, 4.66)	2.20(0.93, 5.25)
Secondary (7-12)	58	152	2.17(1.04, 4.54)	**2.55(1.19, 5.43)**
Post secondary (12+)	18	41	2.50(1.05, 5.98)	**3.47(1.37, 8.78)**
**Time since diagnosed for HIV**				
Less than 6 months	10	50	1.00	1.00
6 months and above	111	235	**2.36(1.16, 4.83)**	**2.26(1.06, 4.85)**
**Type of health facility**				
Hospital	74	206	1.00	1.00
Health center	4	20	0.53(0.53, 1.42)	0.98(0.58, 1.64)

n = 406

### Provision of IPT

**Figure 1:**
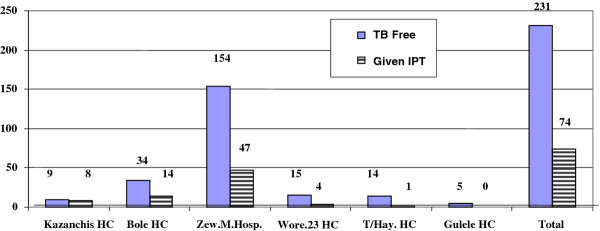
**Provision of IPT in relation to TB free people living with HIV across the health facilties, Addis Ababa City Administration, February 2008**. n = 231.

The proportion of PLHIV who had ever been provided with IPT was 74 of 231 TB free PLHIV (32.0%). This figure varied with health facilities, for example, in Gulele Health Center no one out of five interviewed PLHIV without active TB had been provided with IPT, whereas; in Kazanchis Health Center eight out of nine eligible PLHIV have been provided with IPT (Figure 1).

## Discussion

As the focus for TB control by most public health experts and clinicians in high burden areas is to find active TB cases for prompt care and therapy, the findings in this study for provision of IPT for eligible TB free PLHIV was 32.0%, this means there were considerable missed opportunities. In addition, comparable proportion of interviewed study subjects reported to know (~30%) about the availability of IPT in the respective health facilities. The low coverage of IPT was due to lack of consistency among health care providers towards providing IPT, shortage of Isoniazid and inequitable availability of investigation setups (X-ray, Fine Needle Aspirations, etc.) at the different health facilities to apparently rule out active TB.

Study participants who completed secondary and post secondary education were found to be better informed about the availability of IPT more than those who were not formally educated. These findings may suggest that educated people are more concerned about their disease patterns and seek additional information better than those of less educated. This might be due to better access to printed media than the less educated counterparts beyond other source of information. This finding was also similar with findings in other countries (China, Malawi and Zambia) [11].

In addition, length of being diagnosed for HIV positive and being informed about IPT had a significant association. Those who knew their HIV positive status for more than six months were about two times more likely to be informed about availability of IPT as a package of HIV care than those who were less than six months. This showed that as people stay longer learning about their HIV status, they become more and more aware of their health conditions and opportunistic infections.

In this study females were more informed about the availability of IPT in their health facilities as a package of HIV care for PLHIV than males. This is in contrary to most findings; a study in rural Vietnamese showed males were two times more informed than the females [12]. Our result was also in contrary to a previous finding where higher proportion of males (87%) had some kind of educational attainment than females (75%) indicating better access to printed media in Addis Ababa [13]. However, it is very difficult to make a strong conclusion as the study participants self-select health facilities or it maybe due to men going to private facilities as they are better off in economic status than women.

IPT is one of the key interventions recommended by WHO in 1998 to reduce the burden of TB in PLHIV; yet implementation of IPT had been very low in different countries [14]. The proportion of PLHIV who were free from TB but provided with IPT was only 32.0%. This illustrated the missed opportunities in the prevention of TB in high burden countries such as Ethiopia. However, the proportion of PLHIV in Ethiopia receiving IPT is considerably higher than in most other countries. Globally, only 27,000 PLHIV without active TB were started on IPT (0.1% of the 33 million people estimated to be infected with HIV), almost all of whom were in Botswana [15]. In another study in Italy, for example; the number of people actually starting IPT was very low. This was because of the small number who had a positive TST, contraindications to isoniazid and refusal of the eligible to the offered IPT [16].

Generally, provision of IPT among PLHIV in developing countries is linked to operational problems and Ethiopia is not exceptional. There is no system of doing the TST due to lack of national protocol for provision of IPT for eligible PLHIV, lack of trained personnel who do the TST, lack of supply of the test as well as problems related to administration of the TST and follow up.

This study is limited to primary data sources and lacks supportive evidence from secondary data on the TB/HIV collaborative activities. Interviewer and/or observation bias might not be avoided as all the data collectors were health professionals.

## Conclusions

We conclude that the practice of provision of IPT for PLHIV is high, but there is room for improvement. Strengthening of the implementation of IPT among PLHIV through capturing missed opportunities in all health facilities is recommended.

## Competing interests

The authors declare that they have no competing interests.

## Authors' contributions

AD designed, conducted and analyzed the data as part of his thesis work. GK assisted in the design of the study and conducted analysis of the data. Both contributed in the write up of the manuscript. All authors read and approved the final manuscript.

## Pre-publication history

The pre-publication history for this paper can be accessed here:

http://www.biomedcentral.com/1472-698X/12/2/prepub
